# Maltreatment history, trauma symptoms and research reactivity among adolescents in child protection services

**DOI:** 10.1186/s13034-019-0270-7

**Published:** 2019-03-11

**Authors:** Randall Waechter, Dilesha Kumanayaka, Colleen Angus-Yamada, Christine Wekerle, Savanah Smith, The MAP Research Team, Harriet MacMillan, Michael Boyle, Nico Trocme, Eman Leung, Deb Goodman, Bruce Leslie, Brenda Moody

**Affiliations:** 1 School of Medicine, St. George’s University, St. George’s, West Indies Grenada; 2 Pediatrics & Offord Centre for Child Studies, McMaster University, Hamilton, Canada; 3 McMaster University, Hamilton, Canada

**Keywords:** Child maltreatment, Ethics, Impact of research participation, Risk/reward, Inclusion/exclusion

## Abstract

**Objective:**

There is a well-documented link between child maltreatment and poor health across the lifespan. This provides a strong case for ongoing research with youth involved in the child welfare system to reduce negative outcomes and support resilience while being inclusive of youth voices. However, detailed inquiries about maltreatment history and health consequences may cause re-experiencing of events and psychological distress for study participants. Data that accounts for different contexts, such as severity of maltreatment history and current trauma symptomatology, have been limited in considering the question of potential harms to youth who participate in research—especially longitudinal studies.

**Methods:**

This study compared self-reported impact of research participation against maltreatment history and current post-traumatic stress symptomatology among a randomly selected group of adolescents (< 18 years old) in the child protection service (CPS) system.

**Results:**

Adolescents who report more serious child maltreatment and current trauma symptom severity reported higher scores on distress questions from pre- to post-assessment participation. Critically, participants who were more negatively impacted by study involvement also reported greater benefit from study involvement.

**Conclusion:**

The increase in both negative and positive impact does not shift the risk/reward ratio for participation, as risks alone do not increase for this vulnerable group of CPS involved youth. These results are consistent with previous findings from studies involving non-CPS populations and underlies the importance of empirical data to address the question of change in the risk/reward ratio and what factors might play a role in any change. This information can inform inclusion/exclusion criteria for future research with these vulnerable populations, thereby reducing the risk of distress among study participants.

## Introduction

There is a well-documented link between child maltreatment and poor mental health outcomes such as depression [1–5], suicidality [1, 6, 7], substance abuse [2, 3, 6, 8–12] and posttraumatic stress disorder [4, 5, 8, 11, 13, 14]. There is also a well-documented link between child maltreatment and poor physical health across the lifespan [3, 5]. Ongoing traumatic stress associated with early maltreatment experiences can disrupt neuroendocrione and sympathetic nervous system function, leading to gastrointestinal, gynecological, and cardiopulmonary symptoms, chronic pain, diabetes and obesity [15, 16]. Freyd et al. [17] emphasize the need for multi-disciplinary research with victims to inform policy-makers, increase maltreatment curriculum in medical and mental health service fields, educate the public, and analyze the effectiveness of intervention and prevention efforts.

Given potential poor health outcomes, it is critical to implement policies, laws and programs that prevent maltreatment and to be able to track youth-specific input on outcomes [18, 19]. The United Nations Convention on the Rights of the Child bolsters the belief that research participants should be involved in study design and implementation. Article 12 of the Convention states that youth should be empowered to play a vital role in their own development, as well as that of their communities, through active participation and helping them to learn vital life skills. Youth participants’ viewpoints are significant and important, as only they understand what has happened in their own lives. Achieving a balance between the child’s right to have a voice and right for their information to be protected is crucial in research studies involving child maltreatment [1, 5]. The more investigators inform child participants (and their guardians) about the scientific and social value of conducting the studies, the more likely they are to participate in the duration of the study from beginning to end [5]. In return, research investigators have an obligation to maximize benefits for the individual participant and society, while minimizing risk of harm to the individual, which is often referred to as the risk-reward ratio or a beneficence-nonmaleficence balance [20].

Researchers can meet with resistance from Institutional Review Boards (IRBs) concerned about including vulnerable populations because of the potential negative impact on the participants, when and how to intervene if a participant is experiencing distress, and what are best practices for clinical protocols around reporting suspicions of maltreatment. Researchers and IRBs often rely on personal experience when judging the risk-reward ratio to study participants due to a paucity of objective studies that examine this issue [21]. However, data-driven information is needed that recognizes the important role of youth self-report in capturing each participant’s comprehensive maltreatment history, especially since caseworker knowledge may overlap minimally with youth self-report in areas most difficult to disclose, such as child sexual abuse (CSA) [22]. Abuse is generally a private occurrence, and eyewitnesses, especially to the most egregious acts of abuse, are relatively rare [23]. By necessity, interviews about maltreatment must include sensitive questions [24]. The objective nature of traditional observational research methodologies, wherein the investigators track the natural unfolding of events without intervening, can conflict with the need to assist vulnerable participants who experience distress as a result of research study involvement. Most research has focused on high school and collegiate youth, rather than those who are child welfare system involved.

Findings on the impact of research participation that considers types of violence exposure in particular have been mixed. Although slightly more distressed initially after being surveyed, one study found that university students answering questions about sexual violation had a significantly higher tendency to rate the perceived drawbacks of the study as being few and the benefits of the study as high [25]. Participants answering questions regarding stressful life events had similar ratings of the cost/benefit ratio. In a comparative study of adolescents with a history of CSA who were surveyed in relation to their abuse to a sample of adolescents with no CSA history who were surveyed about their exam experiences, Guerra and Pereda [26] found that sexually abused adolescents reported fewer unpleasant emotions as a result of participation than controls. In an open-ended question regarding how the CSA participants felt while answering the questions, the majority of participants expressed a common notion that the study made them feel good, as it allowed them to express feelings that would help them to cope. Fewer than 10% expressed that they were “feeling bad” as a result of the abuse-related questions [26]. A number of other studies have examined the negative impact (e.g., distress, upset, harm to self/others) and benefits (e.g., study was interesting, self-awareness, willingness to participate in future studies) of research study involvement on children and adolescents [27–30], war veterens [31], and victimized/maltreated adults [32, 33]. Results from these studies indicate that most participants acknowledge benefits from research participation despite mild-to-moderate levels of distress, though, one must be cautious in extending findings from studies of research participation in adults to adolescents. Chu and colleagues carried out a study involving 181 school-aged children with and without trauma histories and found that advantages and disadvantages of their research participation and understanding of informed consent did not vary as a function of trauma exposure [27]. A study of 2312 youth ages 14 to 17 who participated in the National Survey of Children Exposed to Violence found that only 4.5% reported being upset by answering survey questions [28]. Among the 1973 adolescents (13–18 years), 74% enjoyed participation and cited altruism and a greater self-awareness as reasons for participating in the study [29]. A study on posttraumatic stress with 203 injured children and their parents found that 52% of children and 74% of parents were glad they had participated; while 77% of children and 90% of parents felt good about helping others [30].

While the current study focuses specifically on adolescents, a few studies have examined the impact of research involvement among a slightly older population—young adults. A study of female undergraduate University students with childhood history of abuse exposed the participants to procedures directly related to personal trauma experiences and to an arousal-inducing procedure unrelated to individual trauma experiences. One week after participants completed session one of the experiment, only 6% reported that they were unwilling to participate again. This percentage of participants who were unwilling to participate again went up by a small amount after session two and four, but the authors attributed this to factors other than the short term distress that was caused during the study (Carter-Visscher, Naugle, Bell, and Suvak 2003). Another study found that college women with histories of sexual abuse experienced more upsetting feelings than women who had not experienced sexual abuse, but also greater benefits [33]. Benefits to research participation outweighed costs for both women with and without sexual victimization histories.

Using measures and methods suitable for the targeted research population is of particular importance when studying maltreated populations of youth. In designing the Juvenile Victimization Questionnaire (JVC), Hamby et al. [34] took rigorous steps to ensure that their self-report questionnaire would be appropriate for use among a maltreated youth population. They conducted focus groups with researchers, measurement and victimization experts, community organizations, parents, and adolescents to gain feedback on conceptual integrity and appropriateness of the phrases and terminology for use across a youth population [34]. Upon administration of the JVC to a population of youth (10 to 17 years of age) and parents (with children 2 to 9 years old), high amounts of recent victimization were reported (71%) with little confusion from the respondents and little reluctance in answering questions about sensitive material [35].

Chu et al. [27] minimized distress in participants by providing them the opportunity to ask questions before and during the study such as “Do you have to do everything I ask you to do today?”; “Do you have to answer every question I ask?”; “Can you take a break whenever you want to?”; “If you become upset or bored today, what can you do?”; “Do you have to finish the experiment today?”; “Can you stop if you feel like stopping without a ‘good reason’?”; “Can you say ‘pass’ any time you don’t want to do something or don’t want to answer a question I ask?” These questions provided participants with a sense of self-control and helped establish trust between participants and the research team. The authors reported that the risk-reward ratio was positive for the vast majority of participants, with only 1.6% (or 3 out of 186) making negative appraisals of participation [27]. Hasking et al. [29] worked to minimize participant distress by gathering and handing information of mental health resources that could help the participants after the study, if they felt distressed. This procedure was repeated at follow-ups. Their study was reported as a positive experience by 74% of participants [29]. Kassam-Adams and Newman [30] offered research participants access to a counsellor to manage any distress they may have experienced as a result of study involvement. Distress from research participation was only reported by 5% of children and parent participants in that study [30].

Objective evidence about the link between maltreatment experiences or trauma symptomatology and ratings of study involvement is needed to inform methodologies and ethical debates regarding the risk-reward ratio of participating in such studies. Specifically, it is important to understand whether individuals who experienced extreme child maltreatment and/or severe trauma symptomatology are more heavily burdened by study participation. If so, the risk/reward ratio of study involvement for these participants may become negatively skewed, indicating that they should not be involved in such studies given the potential harms. To date, studies examining this question among a population of child welfare-involved youth, especially in the context of current trauma symptoms, are extremely limited. This study addresses this research gap by comparing the self-reported impact of study participation against maltreatment history and current post-traumatic stress symptomatology among a group of randomly selected adolescents (< 18 years old) who were child welfare-involved. The specific aims were to determine if severity of reported child maltreatment history and current trauma symptoms correlate with assessment of study involvement. Based on the results of existing studies, we hypothesized that [36] youth with severe trauma symptoms would rate their participation in the study as more distressing and upsetting, [37] youth who experienced severe child maltreatment would rate their participation in the study as more distressing and upsetting, and [38] youth reporting high levels of distress would also report high levels of benefit from study participation. While few in number, previous studies have shown that participants who report greater distress also report gaining more from study participation [26, 33]. This may be because it gives them an opportunity to share with someone who is listening and is genuinely interested in learning about their experiences.

## Methods

### Participants

We examined self-report of research study participation among a randomly selected group of adolescents (< 18 years old) receiving child protection services (CPS) from a major Canadian urban center. All youth had their own caseworker who was mandated to meet with them on a regular schedule. Data were obtained from a larger research project called the Maltreatment and Adolescent Pathways (MAP) longitudinal study (see [39]. In that study, participants were drawn via a random numbers table from CPS agency-provided master lists of all active caseloads of youth, aged 14–17. Researchers worked with CPS staff members to screen randomly selected youth for study inclusion using predetermined eligibility criteria. Of 1910 referred youth, 1073 were not eligible for study involvement—in most cases because the file was opened and closed during the referral process (62% or 668 of 1073 ineligible referrals). The rest of the 405 ineligible referrals were due to significant developmental delay, being in secure custody, current severe psychiatric health issues, or not being in active contact with CPS care. Of the remaining 837 eligible referred youth, 276 refused to participate in the study, leaving 561 youth, or 67% of the eligible total, involved at the initial testing point of the MAP longitudinal study. It is important to note the relatively small proportion of referred CPS youth in the final sample (561 of 1910 or 31.9%) as a significant limitation of the study. Specifically, our sample is not representative of all CPS-involved youth but those whose cases were more significant to CPS authorities (i.e., open and active longer than a 6 to 12-month referral process) and those who were not such severe cases that contact with the youth was deemed as likely harmful (i.e., secure custody, severe psychiatric illness) or inappropriate for the youth (i.e., severe developmental delay).

A total of 179 youth who scored above the cutoff on the CTQ minimization-denial validity scale and/or the TSCC under-response/hyper-response validity scales (explanations below) were removed from all analyses, leaving a maximum sample size of 382 youth responses on the survey reactivity questions. The exclusion of youth who scored above the cutoff on the CTQ minimization-denial validity scale and/or the TSCC under-response/hyper-response validity scales was necessary to ensure that the youth in our analysis were not underemphasizing or denying their maltreatment history and current trauma or over-emphasizing their current trauma. This denial or overemphasis could significantly skew the study results. Demographic characteristics for the final sample of MAP youth included in the current paper are listed in Table 1. The average age at MAP study entry was 15.8 years (*SD *= 1.04; 46% boys), which included diverse ethnicity (youth-identified ethnicity: 30.4% White only, 26.9% Black only, 26.9% reporting multi-ethnicity, and 15.3% other). The majority of youth (60.9% or n = 342) were Crown Wards, in which biological parents no longer have legal authority of the children. The social worker provided consent for wards of the province (parental rights terminated) and parental consent was obtained for youth living at home, youth aged 16 and above provided their own consent. As such, active consent was obtained as appropriate to the jurisdiction.

**Table 1:** Description of participants included in the current analysis

Variables	Initial test (N = 382) M (SD)
Age in years	15.87 (1.05)
Gender (% male)	48.2%
Self-identified ethnicity	
White	30.4%
Black	26.1%
Other	13.9%
Combination of two or more	29.6%
Child protective services status	
Crown ward (parent rights terminated)	60.7%
Society ward (parent-CPS sharing rights)	13.7%
Interim/temporary care	7.0%
Community family	18.6%
Living status	
Group home	21.5%
Foster home	43.9%
In community with parents/caregivers	28.5%
Other	6.1%
Number of years involved with CPS	5.75 (4.24)
Number of different CPS workers	3.06 (1.60)

### Informed consent

Ethical clearance for the MAP longitudinal study was obtained from CPS agencies and relevant university IRBs. The MAP procedure was to contact the legal guardian (i.e., biological/foster parents and/or the CPS worker) who provided consent for youth under age 16 and when a youth was 16 or reached 16 (via longitudinal study), youth consent was obtained. CPS lawyers were consulted in writing the consent forms. Given the legal requirements of child abuse reporting, youth were told forthright that anything they responded to on the anonymized MAP questionnaires would be kept confidential. The data packets were tagged with a participant ID number. As such, the research assistants never had access to the participants’ responses. The data analyzers only had access to the compiled data, and could not link any single youth identity to the data.

Data was collected electronically, via cellular data connection to a remote server. However, if youth verbally disclosed maltreatment episodes, harm to self, or harm to others to the MAP research assistant during the data collection meeting, those disclosures were reportable. Youth participants were informed of this disclosure distinction both verbally and in the written consent form. In cases of verbal disclosure, the MAP research assistant would inform the CPS worker and contact CPS intake if the worker confirmed that the maltreatment was new or unknown. The MAP research assistant was to contact the study principal investigator and project manager within 24 h of the report, and document the disclosure and all actions taken in a written report. MAP research assistants were also instructed to watch for signs of distress among the youth during all meetings and testing sessions, and not leave the youth if these signs were seen. The research assistants were instructed to seek help from CPS group home staff members or foster home guardians where needed, and call clinicians supporting the project or emergency medical personnel if such assistance was not available. The research assistants had project-supplied cell phones and were also instructed to call the project manager and/or principal investigator for support. No new reports of maltreatment were filed during the full MAP longitudinal study. Youth received a help sheet that listed local resources and 24-h help lines at the end of each session.

### Procedure

After confirming eligibility of the randomly selected youth, CPS caseworkers introduced the MAP study to the youth and sought his/her consent to be called by MAP study researchers to provide more information, schedule an appointment, and obtain final youth consent. Once the CPS worker provided written clearance, MAP study research assistants called the youth to set an appointment. While the IRBs were wary of paying youth for their participation given potential coercion, the researchers argued that the youth, who had experienced victimization in the past, should be paid a minimum wage as a demonstration of respect for their time. As such, participants were paid the existing minimum wage of $7.00 per hour x maximum interview time of 4 h = $28.00. Youth were also given refreshments, and reimbursed for travel to a testing site (community hospital, CPS agency office) if relevant. Youth were given the option of where to be tested, whether at their CPS agency, a community resource center or at home. Most youth (80%) chose home and testing occurred if there was a private room available in the home for conducting the testing.

### Materials

Youth completed batteries of mostly standardized and lab-developed surveys, tests and assessments across time points. For the current analysis, we focused on maltreatment experiences, trauma symptomatology, and assessment of study involvement.

#### Maltreatment

Experiences of childhood maltreatment were assessed via the Childhood Trauma Questionnaire. The CTQ short-form assesses maltreatment via a standard stem (e.g., “While you were growing up…”), rating 28 items on a five-point scale (1 = “never true” to 5 = “very often true”). There are five subscales nested within the CTQ, each consisting of 5 questions: emotional neglect, physical neglect, sexual abuse, physical abuse, and emotional abuse. There are an additional 3 questions that assess the validity of the CTQ (i.e., minimization-denial). Wekerle et al. [39] report 2-week test–retest reliability of the CTQ for a MAP youth subsample (*n *= 52) as moderate, ranging from *r *= .52 to *r *= .70, and internal validity as high, ranging from *r *= .68 to *r *= .92. Wekerle et al. [40] also performed a principal components extraction with varimax rotation using the MAP data to confirm the factor structure of the CTQ with a maltreated population of youth. While the factor structure for CPS males matched the reported five-factor structure, a four-factor structure emerged for females, whereby emotional abuse and physical abuse items co-loaded [40]. For the present report, youth who scored above the cutoff on the minimization-denial validity scale were removed from all analyses.

#### Trauma symptomatology

PTSD symptomatology was assessed via the Trauma Symptom Checklist for Children (TSCC). The TSCC is a 54-item self-report measure that was normalized on teens and is intended to evaluate children who have experienced traumatic events. The TSCC consists of six clinical scales (anxiety, depression, anger, PTSD, dissociation, and sexual concerns) and two validity scales (under-response and hyper-response). Reliability is high (internal consistency is .82–.89) and good convergent, discriminant, and construct validity have been established. Wekerle et al. [39] report moderate 2-week test–retest reliability (*r *= .50) and very high internal validity (r = .97) of the TSCC among a MAP subsample of youth (*n* = 52). For the present report, youth who scored above the cutoff on the under-response or hyper-response validity scales were removed from all analyses.

#### Monitoring youth responses to study involvement

Given the sensitive nature of many of the survey items, in conjunction with the nature of the population of participants, several questions were incorporated into the MAP questionnaire package to measure reactivity to the survey. Specifically, participants were asked to respond to six questions at the end of the questionnaire package using a 7-point [0 (not at all) to 6 (a lot)] scale. Questions included: (1) How interesting did you find these study questions? (2) How distressing did you find these study questions? (3) How clear did you find these study questions? (4) I gained something from filling out this questionnaire? (5) Completing this questionnaire upset me more than I had expected? (6) Had I known in advance what completing this questionnaire would be like for me, I still would have agreed?

## Results

Mean (standard deviation) responses across the six study evaluation questions are presented in Table 2. Responses range from 0 (not at all) to 6 (a lot). Participants found the questions relatively interesting and clear. The mean response score was highest (4.63) for “had I known in advance what completing this questionnaire would be like for me, I still would have agreed.” Importantly, the mean response score was lowest (.91) for “completing this questionnaire upset me more than I had expected.”

**Table 2:** Study evaluation question sample size, mean (standard deviation) ratings: 0 (not at all)–6 (a lot)

	n	Mean (SD)
Q1: How interesting?	381	3.92 (1.49)
Q2: How distressing?	379	2.18 (1.78)
Q3: How clear?	382	4.49 (1.40)
Q4: Did you gain something?	380	3.41 (1.71)
Q5: Questionnaire upsetting?	382	.91 (1.56)
Q6: Still would have agreed?	380	4.63 (1.61)

### Hypothesis 1

Participants with severe trauma symptoms would rate their participation in the study as more distressing and upsetting.

To test this hypothesis, participants were divided into two groups: [36] below the clinical cutoff on all six of the TSCC subscales, and [37] above the clinical cut off on any of the six TSCC subscales. The two groups were then compared across each of the study involvement rating items using an independent samples *t* test (Table 3). The above clinical cutoff group found the study more distressing [*t*(1377) = 3.37, *p *= .001] and more upsetting [*t*(1380) = 2.23, *p *= .028] than the below clinical cut off group. To balance out this higher endorsement of distress and becoming upset by the questionnaire, the above clinical cut off group was more likely to positively endorse gaining something from their participation in the survey compared to the below clinical cut off group, *t*(1378) = 2.43, *p *= .015. The above clinical cut off group also showed a higher mean score than the below clinical cut off group on the final assessment item about still agreeing to complete the questionnaire after knowing what it would be like, but this was only at a trend level.

**Table 3:** Study evaluation mean, (standard deviation), and *sample size* ratings for clinical cut off (below cut off vs. above cut off) on any TSCC subscale

	Below cut off M (SD) *n *=* 296*	Above cut off M (SD) *n *=* 83*	*t*	*p*
Q1: How interesting?	3.85 (1.52)	4.14 (1.37)	1.58	.114
Q2: How distressing?	2.02 (1.71)	2.76 (1.94)	3.37	*.001*
Q3: How clear?	4.55 (1.43)	4.32 (1.27)	1.33	.185
Q4: Did you gain something?	3.30 (1.71)	3.81 (1.68)	2.43	*.015*
Q5: Questionnaire upsetting?	0.81 (1.45)	1.30 (1.87)	2.23	*.028*
Q6: Still would have agreed?	4.56 (1.67)	4.89 (1.33)	1.89	.060

### Hypothesis 2

Participants who experienced severe child maltreatment would rate their participation in the study as more distressing and upsetting.

To test this hypothesis, participants were divided into two groups, [36] below the severe cutoff on all five subscales of the CTQ: emotional neglect, physical neglect, sexual abuse, physical abuse, and emotional abuse (n = 222), and [37] above the severe cutoff on any of the five CTQ subscales (n = 154). While fewer youth met cutoff criteria for severe maltreatment, this was expected as youth have varied family experiences and consequently, different levels of maltreatment. However all the youth were involved in CPS, meaning they had all experienced some form of maltreatment at some point in their past. These two groups were then compared across each of the study involvement rating items using an independent samples t-test (Table 4). The above severe cutoff group found the study more distressing, [*t*(1377) = 2.20, *p *= .028] and upsetting (at a trend level) [*t*(1377) = 1.71, *p *= .087] than the below severe cut off group. To balance out this higher endorsement of distress, the above severe cut off group was more likely to positively endorse that the survey was interesting [*t*(1379) = 2.68, *p *= .008], clear [*t*(1380) = 2.04, *p *= .042], and that they still would have agreed to complete the questionnaire after knowing what it would be like [*t*(1378) = 2.27, *p *= .024] compared to below the severe cut off group.

**Table 4:** Study evaluation mean, (standard deviation), and *sample size* ratings for severe maltreatment cut off (below cut off vs. above cut off) on any CTQ subscale

	Below cut off M (SD) *n *=* 222*	Above cut off M (SD) *n *=* 154*	*t*	*p*
Q1: How interesting?	3.75 (1.53)	4.16 (1.39)	2.68	*.008*
Q2: How distressing?	2.02 (1.71)	2.43 (1.87)	2.20	*.028*
Q3: How clear?	4.61 (1.42)	4.32 (1.34)	2.04	*.042*
Q4: Did you gain something?	3.38 (1.73)	3.45 (1.70)	.39	.699
Q5: Questionnaire upsetting?	0.80 (1.46)	1.08 (1.70)	1.71	.087
Q6: Still would have agreed?	4.48 (1.69)	4.85 (1.46)	2.27	*.024*

### Hypothesis 3

Participants reporting high levels of distress would also report high levels of benefit from study participation.

To test this hypothesis, Pearson’s correlations were run between the distress items (Q2: How distressing? and Q5: How upsetting?) and the benefit item (Q4: Did you gain something?). Question 2 (How distressing?) was significantly correlated with Q4 (Did you gain something?), *r*(1377) = .231, *p *< .001 and Q5 (How upsetting?), *r*(1379) = .383, *p *< .001. However, Question 5 (How upsetting?) was not significantly correlated with Question 4 (Did you gain something?), *r*(1379) = .054, *p *= .290. While these are moderate correlations, it appears that as ratings of being distressed increased among all the participants, ratings of gaining something from the questionnaire also increased. This corresponding relationship was not seen between increased ratings of being upset and gaining something from the questionnaire. It is also important to note that it is impossible to draw any causative inference from these correlations, and that one or more extraneous variables could be playing a role in this relationship.

## Discussion

A number of studies include measures of research impact in an effort to bring objective evidence to the debate about the ethics of asking versus not asking about abuse [37]. The present study addresses this dearth of evidence on child welfare system involved youth by comparing self-reported impact of study participation against maltreatment history and current trauma symptomatology among randomly selected adolescents from the caseload that was receiving CPS services.

Participants above the clinical cutoff for at least one trauma subscale (i.e., anxiety, depression, anger, PTSD, dissociation, sexual concerns) found the study more distressing and upsetting, confirming the first hypothesis. However, those same participants found the study to be more interesting compared to those below the clinical cutoff. Participants who reported experiencing at least one form of extreme child maltreatment (i.e., physical abuse, emotional abuse, sexual abuse, physical neglect, emotional neglect) found the study more distressing than those below the cutoff, partially confirming the second hypothesis. However, those same participants found the study to be more interesting, the questions to be clearer, and they were more likely to report that they would still have agreed to participate in the study after knowing what was involved, compared to those below the cutoff for extreme child maltreatment. There was a significant positive correlation between study distress and benefit of study participation, reaffirming the hypothesis that as the negative impact of study involvement increases, so too does participants’ confirmation that they gained something from their study involvement. This finding was limited to reports of increasing distress in particular.

In summary, CPS-involved adolescents who report more serious child maltreatment and current trauma symptom severity reported more distress and becoming upset because of their involvement in the study. This is consistent with previous findings regarding detailed inquiries about maltreatment history and health consequences causing re-experiencing of events and psychological distress for study participants who experienced maltreatment [20, 21, 29, 42, 43].

Critically, participants who were more negatively impacted by study involvement also reported greater benefit from study involvement. As such, the increase in both negative and positive impact does not shift the risk-reward ratio for participation. It is suggested that a higher level of distress resulting from participation in a study may be a result of increased emotional engagement with the study [44]. In turn, those who feel more connected with the study may be more inclined to perceive the study as positive, despite increased risk of negative emotions elicited by their participation. Consistent with previous findings from studies of research study impact among traumatized (but not CPS-involved) populations, these results indicate that extraordinary precautions are not generally needed for studies with CPS-involved adolescents as the risk-reward balance is favourable [45]. This information can inform inclusion/exclusion criteria for future research with these vulnerable populations.

Future research with adolescents who have a history of maltreatment should implement procedures that will reduce the risk to study participants via: (1) a well-written and clear consent form that explains the study objectives, stipulates freedom to withdraw from the study at any time without having to give a reason [20, 21, 29, 42, 43] and that stipulates the limits to confidentiality (if any); (2) ensuring participants understand that the data they give will be removed from the study at any time following their request [21]; (3) ensuring that robust systems are in place to support any participant who shows signs of distress during or following study involvement; (4) ensuring that all research personnel and collaborators are well-trained and closely supervised by professional psychologists/social workers/healthcare workers; (5) carefully considering the invasiveness of the study methods and ensuring that all measures and questions are necessary to answer well-vetted and important research questions. All of these steps are critical to minimize participation bias and ensure the inclusion of the most severely maltreated children and youth in studies. Investigators must be prepared to deal with the emotional reactions that research participants may experience following very sensitive questions, including debriefing procedures and trained interviewers who look for signs, such as emotional distress, that may indicate a need for clinical intervention [24].

## Limitations

A key limitation of the present study is the small proportion of youth included in the final analysis compared to the number of youth referred for inclusion from all active CPS case files (561 of 1910 or 31.9%). Thus, our sample is not representative of all CPS-involved youth but those whose cases were more significant to CPS authorities and those who were not such severe cases that contact with the youth was deemed as likely harmful or inappropriate for the youth. Further, since most of the youth in the study were in the 14–17 age range, the results may not be generalizable to CPS-involved children and/or youth outside adolescence. Another limitation is the lack of pre-study anxiety level assessment of the participants, which may have affected the study results. Also, responses given by the participants may have been influenced by their desire to meet the assumed expectations of the researcher. Lastly, future research can also focus on chronicity and recency of trauma experiences to conclude if upsetting or reexperincing emotions associated with research participation is greater among youth participants with more severe or more recent traumatic experiences.

## Conclusion

Investigators and ethics boards need to be concerned about including vulnerable populations in research studies that ask potentially distressing questions about past traumas that may place the participants at risk. There has been a rapid increase in the number of studies with vulnerable populations that measure the impact of research involvement. However, very few of these studies have measured the impact of research involvement on maltreated children and youth. We provide evidence that, while the burden of study involvement is higher for youth with a history of extreme maltreatment and youth experiencing severe trauma symptoms, the payoff is also higher. Thus, the risk-reward ratio remains consistent for this vulnerable group. Their involvement in these studies is justified given that participation enables an oft-hidden, marginalized population to have their voices heard and provides findings that can inform otherwise adult-centric research, policy and practice initiatives. This finding is generally consistent with the findings of other studies involving vulnerable populations (e.g., [27–33]). While participants may become distressed when hearing or talking about experiences that have been traumatic, difficult, confusing or frightening, the process through which this expression of emotion is planned for, acknowledged and managed is critical [43]. Given the limitations of this study, especially the exclusion of the most severely impacted CPS-involved youth, future studies should attempt to examine this especially vulnerable population to determine whether the risk-reward ratio is also balanced, though potentially shifted higher among this group. We hope this study also contributes to and encourages a growing trend of using empirical data to inform ethical questions about participation of potentially vulnerable groups in research studies. These groups are often most in need of effective interventions and should therefore be given the opportunity to participate in studies as long as the risk/reward balance is stable.
